# Preparation and Performance of Leather-Finished Plywood

**DOI:** 10.3390/polym16182587

**Published:** 2024-09-13

**Authors:** Yuanyuan Zou, Ziyi Yuan, Yuxin Lu, Xiaoqian Liu, Chuxuan Chen, Lu Fang

**Affiliations:** 1 College of Furnishings and Industrial Design, Nanjing Forestry University, Nanjing 210037, China; yuanziyi@njfu.edu.cn (Z.Y.); luyuxin@njfu.edu.cn (Y.L.); tangren2022123@163.com (X.L.); 13790862589@163.com (C.C.); fanglu@njfu.edu.cn (L.F.); 2 Co-Innovation Center of Efficient Processing and Utilization of Forest Resources, Nanjing Forestry University, Nanjing 210037, China

**Keywords:** leather, EVA film, intermediate layers, overlaying performance

## Abstract

In order to achieve batch production, we propose a simple and fast method to prepare leather-finished plywood. In this study, ethylene–vinyl acetate was selected as the intermediate layer to prepare EVA/polyurethane (PU) leather composites. ESEM, tensile property test and compressive property test were used to characterize the microstructure and physical-mechanical properties of the composites. The response surface method (RSM) was also used to explore the relationship between hot pressing temperature, hot pressing pressure and hot pressing time. The significance of the factors and the interactions between the two factors were determined by ANOVA, with the most significant effect being that of the temperature. The theoretical optimal hot pressing process conditions were calculated by the regression equation as a temperature of 124.4 °C, a time of 200 s and a pressure of 1.3 MPa. The surface bond strength of the test specimen measured under this condition was 1.89 MPa, it has good finishing properties and the impregnation peel strength and surface bond strength met the requirements of GB/T 15104-2021.

## 1. Introduction

Custom furniture can be manufactured with adjustable size, in various combinations and with good space utilization to meet the personalized needs of consumers [1,2,3,4,5]. Finishing materials with wood-based panels have evolved rapidly to satisfy consumer demand for well-designed custom furniture of high quality. Mainstream finishing materials include decorative paper, paint, veneer and plastic film, predominantly [6,7,8,9]. Among the many decorative materials, decorative paper is the most widely used [10]. It is used mostly for the manufacture of custom furniture. The decorative paper is generally printed by impregnating plain paper with melamine formaldehyde (MF) and other aldehyde adhesives and then dried [11,12]. With the gradual improvement of green policies nationally, the preparation of VOC-free organic polymer resins such as water-based polyurethane and polyacrylate as impregnating resins for aldehyde-free decorative paper has received widespread attention in recent years. Long Ling’s team at the Chinese Academy of Forestry Sciences (CAFS) targeted the synthesis of small particles of water-based polyurethane/water-based polyacrylate [13,14]. This team developed a non-formaldehyde resin with good permeability which does not stick to the steel roller during roller pressing. The aldehyde-free decorative paper prepared with this resin has good flexibility and shows strong adhesion to various wood-based panel substrates. Decorative adhesive film is an ideal material for wood-based panel materials. It is multi-colored and has excellent performance, which ensures the rich appearance of the artificial board and effectively enhances the resistance of the board surface to wear, stain and water vapor [15,16,17,18]. However, decorative paper is hard to the touch and poorly breathable, which can lead to a cold sensation.

Leather, whether natural or artificial, has the characteristics of softness, comfort, breathability, durability and wear resistance, which can give people a warm feeling [19,20,21]. The application of leather in furniture has a long history, not only as a decorative surface material, but also as an integral structural component of furniture [22]. Classic leather furniture, including the Barcelona Chair and Wassily Chair, exploited the unique characteristics of leather. Currently, leather and furniture are combined mainly by wrapping, winding and weaving methods [23,24]. The different combinations meet different furniture designs and styles but are not conducive to the large-scale production of custom leather furniture. A flat press to laminate leather onto the surface of wood-based plywood can be used for the large-scale production of leather custom furniture. However, due to the special structural characteristics of leather, traditional adhesives are not suitable for the preparation of leather-decorated plywood. Currently, some enterprises use the technology of cold pressing flat lamination to fabricate leather-finished plywood with white latex as adhesive. However, the leather-finished plywood has low surface bonding strength and poor quality, and the finishing material can be easily peeled off from the substrate during use. Gauthier et al. [25] provided a new method for preparing leather-finished plywood using the flat press with impregnated thermosetting resin cellulose sheets as adhesive. This process effectively prevents detachment from the substrate due to leather shrinkage during post-processing. However, the preparation of adhesive in this method is complex and expensive.

Recent studies proposed the use of intermediate layers to solve the problem of bonding between different materials. Polyethylene (PE), polypropylene (PP), ethylene–vinyl acetate copolymer (EVA) and other thermoplastic films are widely used as intermediate layers due to their environmental friendliness, flexibility, water resistance and processability [26,27]. Guo et al. [28] grafted maleic anhydride (MAPE) onto PE molecular chains and used MAPE as an intermediate layer, which resulted in strong adhesion between the decorative wood veneer and wood–plastic composites. Fang et al. [29] used PE film as the middle layer and decorative veneer as the finishing material to finish a wood-based panel, and the surface gluing strength of the decoration plywood obtained was ≥0.5 MPa, with good finishing effect. Zhang et al. [30] used EVA as an intermediate layer to prepare EVA-film-reinforced decorative veneer with significantly increased flexibility and transverse tensile strength. The surface bonding strength after finishing reached 1.28 MPa. The impregnated peel length was 0 mm. Thus, the utilization of veneer was greatly improved. Among the many thermoplastic film materials, EVA has the lowest melting temperature (about 85 °C), which can effectively reduce the damage to the leather surface during the gluing process.

Artificial leather has a similar appearance to natural leather but with brighter colors and lower cost. Therefore, polyurethane (PU) leather was selected as the decorative material, and the EVA film was used as the intermediate layer to finish the plywood. The effects of process parameters such as hot pressing temperature, time and pressure on the performance of the leather-decorated plywood were systematically analyzed.

## 2. Materials and Methods

### 2.1. Materials

PU leather(Guangzhou Dongyi Leather Co., Ltd., Guangzhou, China) with dimensions of 200 mm × 200 mm × 1.2 mm. The surface texture of the PU leather was the Nappa pattern, and the back was plush. Manchurian Ash (*Fraxinus mandshurica*) decorative veneer (Deqing Meilun Decoration Materials Co., Ltd., Huzhou, China), with dimensions of 200 mm × 200 mm × 0.5 mm. Impregnated paper (Linyi Haisen Wood Industry Co., Ltd., Linyi, Shandong, China) with dimensions of 200 mm × 200 mm × 0.2 mm. EVA film (Fujian Furong New Materials Co., Ltd., Fuqing, Fujian, China) with a thickness of 0.2 mm and a density of 0.91 g/cm^3^ was used for reinforcement. Poplar plywood (Shanghai Xinban Wood Industry Co., Ltd., Shanghai, China) measuring 200 mm × 200 mm × 15 mm in size, 0.56 g/cm^3^ in density and 8~10% in moisture content. 

### 2.2. Preparation of PU-Leather-Finished Plywood

**Figure 1:**
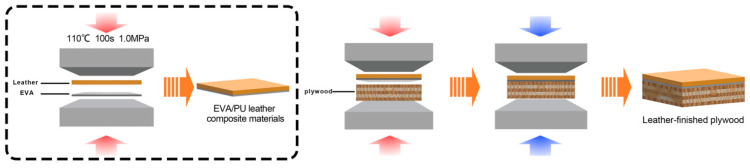
Manufacturing process of PU-leather-finished plywood.

The PU decorative leather was laminated to the plywood surface under different process conditions, and the lamination process is shown in Figure 1. The Box–Behnken design in the response surface methodology (RSM) was used to analyze the effect of three process parameters, hot pressing temperature (A), hot pressing time (B) and hot pressing pressure (C), on the leather-finished plywood using surface bonding strength (Y1) and impregnated peel strength (Y2) as dependent variables. The experimental design was coded at the factor level, as shown in Table 1. Each group of tests was repeated three times.

### 2.3. Characterization

#### 2.3.1. Decorative Leather Performance Tests

- Compression performance. First, the PU leather specimen with a specification of 35 mm × 35 mm was prepared. Second, the leather specimen was spread on the specimen table of the mechanical testing machine and loaded. During the test, the load was increased from 0 N to 1000 N. The changes in the thickness of the loaded specimen were recorded every 0.1 s. The maximum displacement was recorded when the load was 1000 N. Six specimens were selected for testing under each respective test level, and the results were averaged.
- Tensile performance. Based on the standard GB/T 16799-2018 [31] “leather for furniture” preparation specifications for 25 mm × 100 mm PU leather specimens, the displacement was controlled and varied uniformly during the test. The displacement and tensile force were recorded every 0.1 s until the specimen was damaged. The maximum load during the damage was recorded. Six specimens were selected under each test level, and the results were averaged.

#### 2.3.2. Overlaying Performance Test

- Impregnation peel performance. Based on the standard GB/T 17657-2022 [32] “test methods of evaluating the properties of wood-based panels and surface decorated wood-based panels” preparation specifications for 75 mm × 75 mm leather-finished plywood specimens, firstly, the specimens were impregnated in hot water (63 ± 3) °C for 3 h and then dried in an oven at (63 ± 3) °C for 3 h. The peel length of the adhesive layer between the veneer and the substrate was recorded. Six specimens were selected for testing under each respective test level, and the results were averaged.
- Surface bonding strength. Based on the standard GB/T 17657-2022 [32], specimens of leather-finished plywood with specifications of 50 mm × 50 mm were prepared. They were tested with a mechanical testing machine to determine the maximum damage load. Six specimens were selected for testing under each test level, and the results were averaged.
- Microscopic morphology of the gluing interface. The leather-finished plywood specimens were cut to a size of 5 mm × 3 mm. After gold spraying of the cross-section, the gluing interface between the decorative leather, the EVA film interlayer and the plywood substrate was analyzed via environmental scanning electron microscopy (ESEM).

## 3. Results and Discussion

### 3.1. Analysis of Tactile Properties of PU Leather

Tactile properties are important for evaluating materials for surface decoration. In this study, the softness, fullness and elasticity of PU leather were quantitatively evaluated by testing the form variables, tensile strength and tensile fracture energy of several typical decorative materials via compression and tensile experiments.

#### 3.1.1. Compression Performance of PU Leather

The deformation capacity of the material under low compressive load can partially simulate the flexibility determined by subjective perception based on touch or pressing the surface of the material. Usually, a larger form variable represents better fullness and flexibility [33]. Since the thicknesses of the selected leather, veneer and impregnated paper were not uniform, the form variables needed to be normalized. In this study, the ratio of the form variable to the initial thickness (compression coefficient) was used to evaluate fullness and softness.

**Figure 2:**
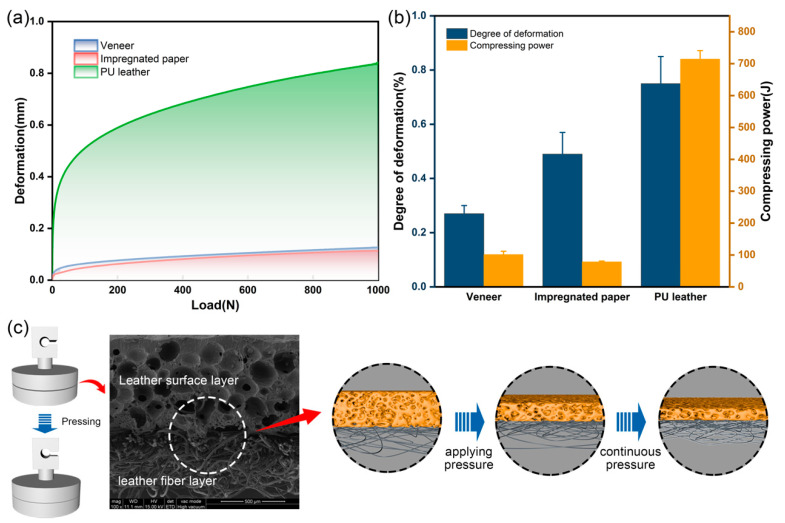
Compression performance test of different materials: (**a**) deformation of PU leather, veneer and impregnated paper; (**b**) degree of deformation and compressing power of three materials; (**c**) microstructural changes in the PU leather during compression.

Figure 2a presents the deformation curves of the three decorative materials, PU leather, veneer and impregnated paper, under pressure. In the initial stage of compression, the deformation of all three materials showed rapid growth. The slope of the deformation–load curve reflects the degree of deformation in different materials under compression load. The greater the slope of the curve, the greater the deformation of the material [34]. In this study, the slope of the linear segment was analyzed. The form variable showed a stable linear relationship with the load when the compressive load was 10 to 20 N. The fitting results showed that the slope of the curve for PU leather was the largest, and, therefore, the softness was the best. As the load continued to increase, the slope of the curve gradually decreased and stabilized for the three materials. As shown in Figure 2a,b, when the compressive load was fixed at 1000 N, the deformation variable of PU leather reached 0.88 mm and the compression coefficient was 0.73, which was substantially higher than that of veneer and impregnated paper. Thus, leather has obvious advantages over other materials in terms of fullness. We determined the compression power of PU leather, veneer and impregnated paper by integrating the deformation–load curves in Figure 2a. The higher the compression power, the better the softness of the material [35]. As shown in Figure 2b, the compression powers of PU leather, veneer and impregnated paper were 714.48 J, 101.54 J and 78.69 J. These data further indicate that the softness of PU leather was significantly better than that of other decorative materials such as veneer and impregnated paper.

The excellent fullness and softness of PU leather are closely related to its organizational structure. The microstructural features are shown in Figure 2c. PU leather consists of a foam surface layer and a fiber base layer. A large number of pores and large pore structures exist in the leather foam surface layer. These structures ensure that the material has sufficient deformation space during compression. At the same time, the fiber layer at the bottom of the leather is flexible and has a loose tissue structure. Therefore, the deformation behavior of the leather is less restricted during the compression process. With the gradual increase in compression load, the loose pore structure and fiber structure in the leather gradually change to a dense state, and the rate of the increase in deformation also tends to stabilize.

#### 3.1.2. Tensile Properties of PU Leather

**Figure 3:**
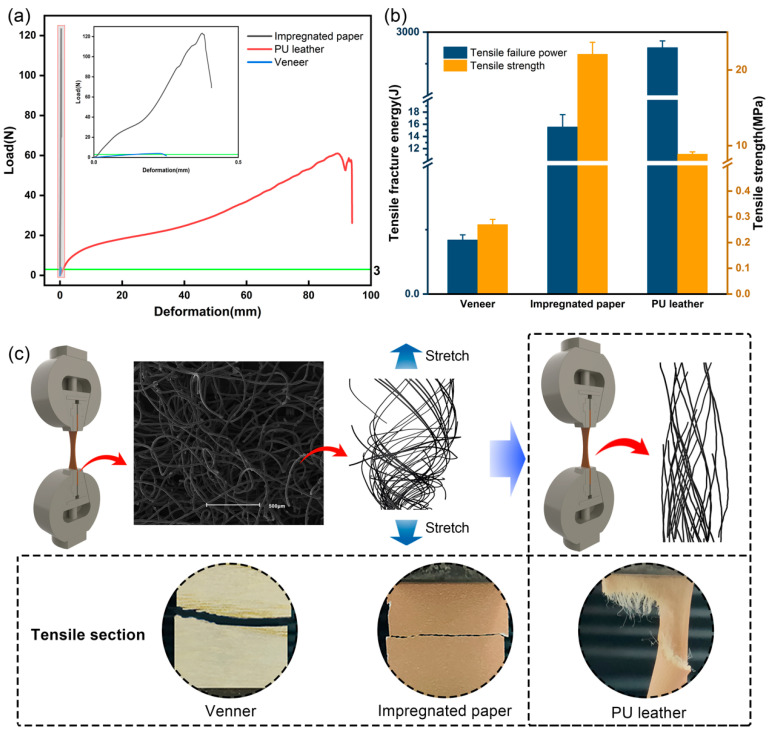
Tensile performance test of different materials: (**a**) load–deformation curves of PU leather, veneer and impregnated paper; (**b**) tensile fracture energy and tensile strength of three materials; (**c**) microstructural changes in the PU leather during tensile and fracture tests and cross-sections of three materials.

Tensile performance indicators include tensile strength, tensile fracture energy and others, which contribute to effective tactile evaluation of leather [36,37]. The tensile properties of the three decorative materials were tested separately, and the results are shown in Figure 3. Under the action of tensile force, PU leather showed a completely different deformation trend and fracture morphology than other materials. As shown in Figure 3a, the three materials yielded significantly different deformation variables in the initial stage of stretching. When the load was 3 N, the deformation variables of PU leather, veneer and impregnated paper were 0.91 mm, 0.16 mm and 0.02 mm. This indicates that a very small force can cause a large strain on the PU leather. The PU leather has a high degree of softness. Further analysis (Figure 3a) reveals that all three materials were in the elastic deformation stage when the load increased from 0 to 3 N. The slope of the straight line in this stage can be used to measure the magnitude of elasticity. As shown in Figure 3a, the slope of PU leather was significantly lower than that of veneer and impregnated paper and inseparable from that of the interwoven fiber structure. Initially, the fibers in PU leather are bent and loose (Figure 3c). When the fiber is stretched, it becomes taut, resulting in larger deformation. However, the fibers at this stage exhibit mainly changes in spatial structure, resulting in a low slope. When the load is increased, the flexibility is poor due to the small thickness of veneer [38]. As shown in Figure 3b, the veneer fractured first under the same tensile conditions. Its calculated transverse tensile strength was only 0.27 MPa. Among the three decorative materials, the impregnated paper had the highest tensile strength of 22.09 MPa, mainly due to the presence of MF resin in the impregnated paper, and most of the resin was in a pre-cured state. The elongation at break of both veneer and impregnated paper is very low. As shown in Figure 3c, their fracture surfaces are relatively smooth due to predominantly brittle fracture. The deformation of PU leather gradually increased throughout the stretching process, and its elongation at break reached 91.74 mm, which was much higher than that of veneer and impregnated paper. These findings demonstrate the advantages of leather in terms of softness. In addition, under tensile fracture, the PU leather cross-section was rougher, with obvious neck shrinkage during the stretching (Figure 3c). This phenomenon is attributed to the large number of fillers and adhesives between the fibers of PU leather. During the tensile test, the fibers cannot be perfectly aligned in a straight line in the direction of the tensile force. In the later stages of stretching, the fibers are gradually closely combined, leading to narrowing of the tensile section of the leather at the macroscopic level.

Tensile fracture energy is the energy required to pull off the material. The magnitude of tensile fracture energy is related not only to tensile strength but also to elongation. It can also be used to evaluate the feel of the material. The load–deformation curves of PU leather, impregnated paper and veneer were integrated, and their tensile fracture energies were 2877.34 J, 15.56 J and 0.21 J, respectively (Figure 3b). This further indicated that the softness and elasticity of PU leather were significantly better than those of other materials.

#### 3.1.3. Effect of EVA Compound on the Tactile Sensation of Leather

**Figure 4:**
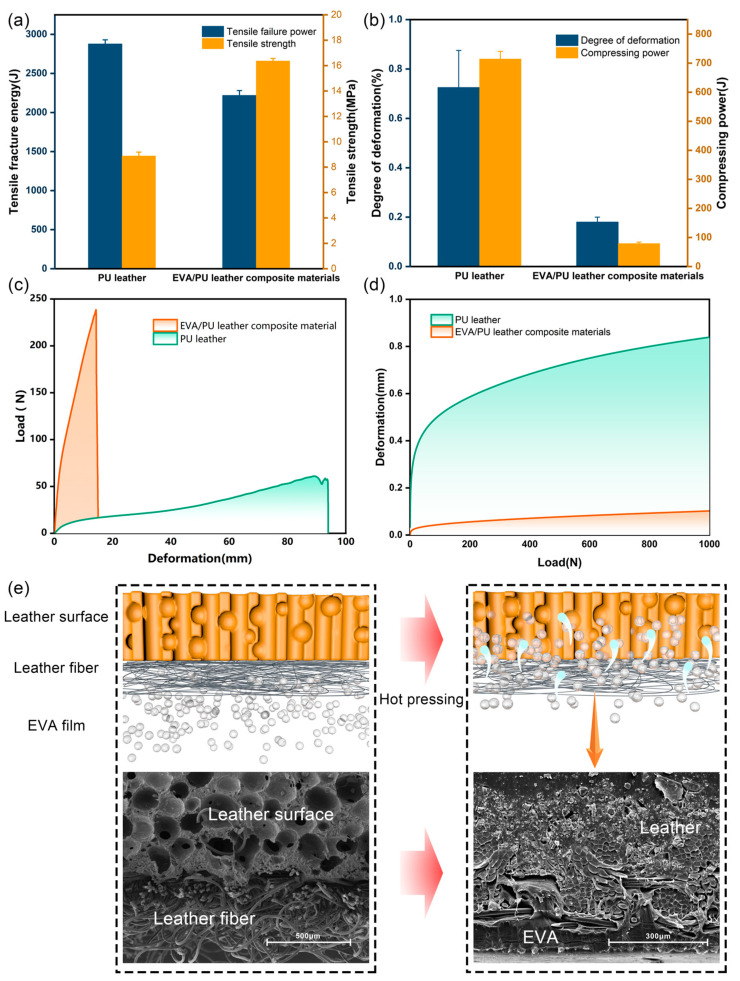
Effect of EVA on the tensile and compressive properties of leather: (**a**) tensile fracture energy and tensile strength of PU leather and EVA/PU leather composite materials; (**b**) degree of deformation and compressing power of PU leather and EVA/PU leather composite materials; (**c**) variation of deformation and load of two materials during tension; (**d**) variation of deformation and load of two materials during compression; (**e**) microstructural of leather before and after EVA lamination.

In order to enhance the stability of the gluing between the PU leather and man-made panel, the leather with an EVA interlayer was prepared for lamination. As EVA film exhibits good flexibility, the introduction of the middle layer significantly improves the tensile strength of PU leather. As shown in Figure 4a, the tensile strength of EVA/PU leather composite materials reached 16.37 MPa, which was 84.14% higher than the original.

By analyzing the compression performance of EVA/PU leather composites, it was found that the introduction of EVA reduced the tactile comfort of PU leather. As shown in Figure 4b, the deformation of EVA/PU leather composites was significantly decreased throughout the compression. Further analysis of the linear section of the curve (10 N to 20 N) and the overall compression characteristics was performed. When PU leather was compounded with EVA, the compression coefficient and compression power were significantly reduced, suggesting a decrease in the softness and fullness of the leather as the molten EVA enters the pores of the leather under pressure during hot pressing. The deformation space of the material is reduced and the fiber activity is restricted (Figure 4e). As the loose structure of the leather is filled, its tensile behavior is also changed and its fracture growth rate and tensile fracture energy are significantly reduced.

### 3.2. Performance Analysis of Leather-Finished Plywood

EVA/PU leather composites were used to decorate poplar plywood. The effects of hot pressing temperature, time and pressure on the surface bonding strength and impregnation peeling of the decorative plywood were investigated using the RSM. The results are shown in Table 2.

During the preparation of leather-finished plywood, the EVA interlayer is melted again to ensure tight adhesion between the leather and the plywood substrate. As shown in Table 2, the minimum surface bonding strength of the leather veneer was 0.98 MPa, which was much higher than the minimum surface bonding strength (≥0.40 MPa) specified in GB/T15104-2021 [39]. The results based on the RSM were analyzed by Design-Expert 13.0 to obtain the regression equation Y = 1.70 + 0.29A + 0.11B + 0.17C + 0.06AB + 0.11AC − 0.20A^2^ − 0.17B^2^ − 0.23C^2^. As shown in Table 3, the model was highly significant (*p* < 0.01), and the coefficient of determination (R^2^) obtained by dividing the regression sum of squares by the total sum of squares was 0.98. This indicates that the model adequately fits the experimental data. It can be used to determine the optimal process parameters, and in the analysis and prediction of their effect on the surface bonding strength of decorative panels.

**Figure 5:**
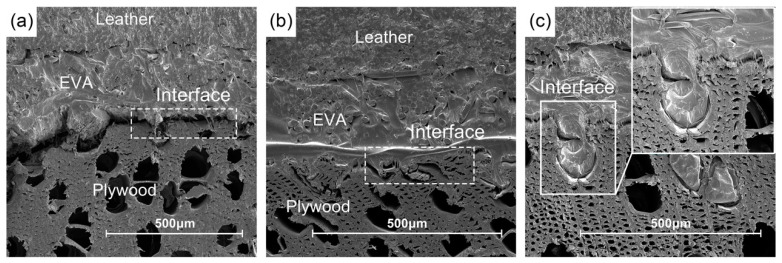
Bonding interface microstructure of the decorative veneer: (**a**) 95 °C, 50 s, 1.0 MPa; (**b**) 125 °C, 150 s, 1.0 MPa; (**c**) 110 °C, 250 s, 1.5 MPa.

Based on the results of the ANOVA, the hot pressing temperature, pressure and time had significant effects on the finishing properties. The effect of hot pressing temperature was the most significant among all factors as it affects the viscosity and fluidity of EVA. When the hot pressing temperature is higher than the melting temperature of EVA, the molecules penetrate into the porous structure of poplar plywood under pressure and form a mechanical interlock with the substrate (Figure 5). When the hot pressing temperature increased from 95 °C to 110 °C, the fluidity of the EVA increased and, further, a glue-nail structure was formed between the EVA and plywood. The surface bonding strength of the veneer was significantly improved. However, due to the viscosity of EVA, a further increase in temperature led to a leveling off of the bonding strength. When the hot pressing temperature was 125 °C, the surface bonding strength reached a maximum of 1.83 MPa, which was less of an improvement compared with the medium level.

**Figure 6:**
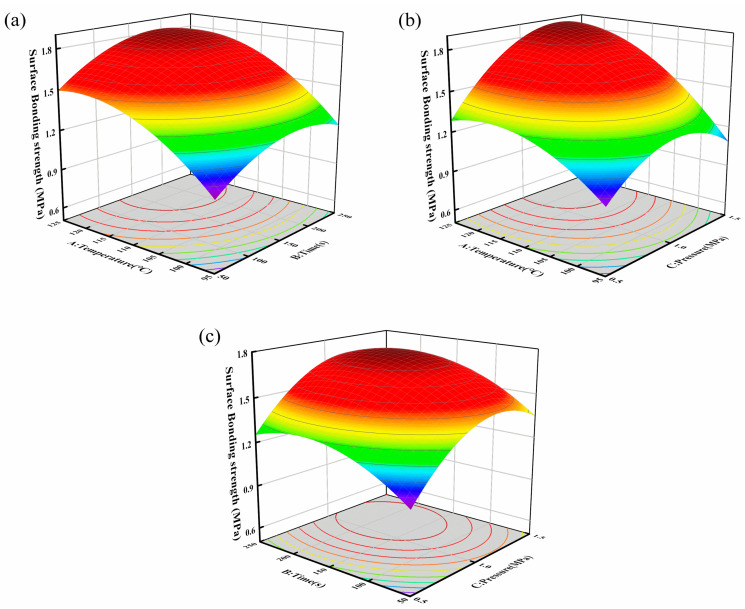
The three-dimensional plots of the response surface: (**a**) the interaction between hot pressing temperature and time; (**b**) the interaction between hot pressing temperature and pressure; (**c**) the interaction between hot pressing time and pressure.

Hot pressing pressure is another important factor affecting the adhesion. When the hot pressing time and temperature were set to medium or high levels, the surface bonding strength of the specimens increased significantly when the pressure was increased from 0.5 MPa to 1.5 MPa. The EVA middle layer and PU leather in the first composite formed a strong adhesive bond. During the secondary melting process, if the pressure is too low, the adhesive cannot effectively penetrate into the pores of the plywood substrate. As shown in the three-dimensional plots of the response surface, the interaction between hot pressing temperature and pressure has a significant effect on the surface bonding strength of veneer sheets. As shown in Table 2, increasing either the hot pressing temperature or the pressure improved the adhesive strength under short durations (50 s). The response surface diagram representing the interaction between temperature, pressure and time during hot pressing is shown in Figure 6. When the hot pressing time was fixed, the density of the contour did not change significantly with increasing temperature. The density of contours along the A-factor (temperature) moving toward the peak was higher than under the hot pressure, which further indicates that the hot pressure temperature contributes strongly to the effect. Under the same hot pressure, extending the time drives additional EVA to penetrate into the plywood and improve the surface bonding strength at the same time. However, the effect of hot pressing time on surface bonding strength is relatively weaker than that of pressure and temperature due to the viscosity of EVA.

Using a regression formula, the theoretical parameters representing the optimum process were obtained, including a temperature of 124.4 °C, a time of 200 s and a pressure of 1.3 MPa. The leather-finished plywood was prepared under these optimum conditions. The surface bond strength of the veneer was 1.89 MPa, with an error of 1.56% between the measured and theoretical values. The test results are stable, reasonable and reliable.

The specimens were subjected to immersion peel test to evaluate their water resistance. As shown in Table 2, among the 17 test groups, only 2 groups showed partial peeling of the prepared veneer panels. The first group showed peeling at 125 °C, 150 s and 0.5 MPa. The second group showed peeling at 95 °C, 150 s and 0.5 MPa. The immersion peel length appeared to be higher in one of the groups at a lower temperature. With the increase in temperature, the EVA molecules were activated and penetrated the substrate to form an adhesive nail structure, resulting in higher resistance of the decorative panels to the hot and humid environment. By increasing the pressure without changing other process conditions, the water resistance of the veneer was significantly improved. When the pressure is low, most of the EVA is retained in the leather fibers and the actual penetration into the plywood substrate is reduced, resulting in poor adhesion of the leather to the substrate. The specimens showed uneven peeling when the veneer was treated with moist heat. As the pressure increased, additional EVA penetrated into the plywood (Figure 5c). This improved the bonding interface between the EVA and the poplar plywood. The average impregnated peel length of the PU-leather-finished plywood produced under the above conditions was very small, which met the requirements of GB/T 15104-2021 [39] (≤25 mm) and resulted in an acceptable outcome.

## 4. Conclusions

Leather has superior elasticity, fullness and softness compared with common finishes such as wood. It meets the basic requirements of veneer finishes. Before preparing leather-finished plywood, the leather was pre-laminated with the EVA film. The tensile strength of the leather was significantly increased when laminated with an EVA interlayer, but its tactile comfort was slightly decreased. Then, the leather/EVA composite was used as the surface decorative material for plywood. The optimum preparation conditions for the leather-finished plywood were a hot pressing temperature of 124.4 °C, hot pressing time of 200 s and hot pressing pressure of 1.3 MPa. The surface bonding strength of the specimens was measured at 1.89 MPa under these conditions. The results of the analysis show that hot pressing temperature and pressure and their interaction had a significant effect on the surface bonding strength of the veneer. The EVA film can be used as an intermediate layer for the preparation of decoration plywood without reapplication of adhesive, and it is possible to achieve rapid and highly efficient production with consistent quality. All of the leather-finished panels passed the type II immersion peel test and can be used in an indoor humid environment.

## Figures and Tables

**Table 1:** Leather veneer plywood preparation hot pressing processing parameters.

Levels	Factors		
	Temperature (°C)	Time (s)	Pressure (MPa)
−1	95	50	0.5
0	110	150	1.0
1	125	250	1.5

**Table 2:** Experimental design and responses for RSM.

Run	Level			Impregnation Peeling Properties/mm	Surface Bonding Strength/MPa
	A	B	C		
1	0	0	0	0	1.67
2	0	0	0	0	1.69
3	1	−1	0	0	1.56
4	0	0	0	0	1.75
5	−1	1	0	0	1.14
6	−1	0	1	0	1.16
7	1	0	−1	1.03	1.21
8	0	1	−1	0	1.31
9	0	0	0	0	1.65
10	1	0	1	0	1.83
11	1	1	0	0	1.79
12	0	1	1	0	1.59
13	−1	−1	0	0	0.99
14	0	−1	−1	0	1.05
15	0	0	0	0	1.70
16	−1	0	−1	4.25	0.98
17	0	−1	1	0	1.29

**Table 3:** ANOVA for experimental results.

Source	Sum of Squares	df	Mean Squares	F-Value	*p*-Value	Significance
Model	1.4284	9	0.1587	34.36	0.0001	**
A	0.5618	1	0.5618	121.63	<0.0001	**
B	0.1105	1	0.1105	23.91	0.0018	**
C	0.2178	1	0.2178	47.16	0.0002	**
AB	0.0016	1	0.0016	0.35	0.5746	-
AC	0.0484	1	0.0484	10.48	0.0143	*
BC	0.0004	1	0.0004	0.09	0.7771	-
A2	0.1195	1	0.1195	25.88	0.0014	**
B2	0.0992	1	0.0992	21.48	0.0024	**
C2	0.2198	1	0.2198	47.60	0.0002	**
Residual	0.0323	7	0.0046			
Std. Dev.	0.0267	3	0.0089	6.26	0.0544	
C.V. %	4.74					
Adjusted R^2^	0.9779					

Note: ** very significant (*p* < 0.01); * significant (0.01 < *p* < 0.05); - not significant (*p* > 0.05).

## Data Availability

The original contributions presented in the study are included in the article, further inquiries can be directed to the corresponding author.
